# The Role of RNA Sensors in Regulating Innate Immunity to Gammaherpesviral Infections

**DOI:** 10.3390/cells12121650

**Published:** 2023-06-17

**Authors:** Huirong Zhang, Praneet K. Sandhu, Blossom Damania

**Affiliations:** Department of Microbiology and Immunology, Lineberger Comprehensive Cancer Center, Center for AIDS Research, University of North Carolina at Chapel Hill, Chapel Hill, NC 27599, USA

**Keywords:** KSHV, EBV, antiviral response, innate immunity, IFN, RNA sensor, RLR, RIG-I, MAVS, MDA5, TLR3, TLR7/8

## Abstract

Kaposi’s sarcoma-associated herpesvirus (KSHV) and the Epstein–Barr virus (EBV) are double-stranded DNA oncogenic gammaherpesviruses. These two viruses are associated with multiple human malignancies, including both B and T cell lymphomas, as well as epithelial- and endothelial-derived cancers. KSHV and EBV establish a life-long latent infection in the human host with intermittent periods of lytic replication. Infection with these viruses induce the expression of both viral and host RNA transcripts and activates several RNA sensors including RIG-I-like receptors (RLRs), Toll-like receptors (TLRs), protein kinase R (PKR) and adenosine deaminases acting on RNA (ADAR1). Activation of these RNA sensors induces the innate immune response to antagonize the virus. To counteract this, KSHV and EBV utilize both viral and cellular proteins to block the innate immune pathways and facilitate their own infection. In this review, we summarize how gammaherpesviral infections activate RNA sensors and induce their downstream signaling cascade, as well as how these viruses evade the antiviral signaling pathways to successfully establish latent infection and undergo lytic reactivation.

## 1. Introduction

KSHV (also known as human herpesvirus 8, HHV8) and EBV (also known as human herpesvirus 4, HHV4) are members of the gammaherpesvirus family and these viruses are associated with multiple human malignancies. KSHV is the etiological agent of Kaposi’s sarcoma (KS), in addition to two B-cell-derived malignancies: primary effusion lymphoma (PEL) and multicentric Castleman’s disease (MCD) [1,2]. More recently, KSHV has also been implicated as a causal agent for osteosarcoma [3]. EBV is linked with B cell lymphomas and epithelial cell carcinomas. They include, but are not limited to, Burkitt’s lymphoma (BL), Hodgkin’s lymphoma (HL), non-Hodgkin lymphoma (NHL), nasopharyngeal carcinoma (NPC) and gastric carcinoma (GC). In addition to its association with human tumors, EBV is also linked to several autoimmune diseases such as systemic lupus erythematosus (SLE) and multiple sclerosis (MS) [4,5,6,7,8].

EBV and KSHV are oncogenic double-stranded DNA (dsDNA) viruses with both viruses exhibiting two distinct phases of their life cycles: latency and lytic replication. During latency, the KSHV genome is replicated as a circular episome by the cellular DNA polymerase and only expresses a limited set of latency-associated proteins and pre-microRNAs [9]. Similarly, EBV also maintains its genome as a latent episome in the nucleus of the host cell and expresses a small group of viral proteins and a number of viral noncoding RNAs including EBV-encoded RNAs (EBERs) and BamHI-A rightward transcripts (BARTs). Furthermore, EBV establishes distinct types of latency programs (III-II-I-0) in different cell types based on specific latent gene expression patterns [8]. Under certain conditions, both viruses are able to reactivate and enter the lytic cycle. During lytic reactivation, all viral genes are transcribed, viral DNA is amplified, progeny virions are produced, and this eventually leads to the death of reactivating cells [10,11]. As there are more viral proteins and noncoding RNAs induced during lytic replication, the host’s immune responses tend to be more pronounced during lytic infection compared to latency.

The innate immune response triggered by nucleic acid recognition plays an important role during viral infection. This process is initiated by nucleic acid sensors that recognize foreign DNA and RNA, such as viral genomes. Upon sensing the viral nucleic acids, the DNA/RNA sensors and their signaling cascades are activated to produce type I interferons (IFNs) and proinflammatory cytokines, which establish an antiviral state and inhibit viral infection. Several host DNA and RNA sensors have been reported to limit KSHV or EBV infection, including endosomal Toll-like receptors (TLRs), cytosolic DNA and dsRNA sensors cyclic GMP–AMP synthase (cGAS) and retinoic acid-inducible gene I protein (RIG-I)-like receptors (RLRs) [12,13]. RLRs encompass two major dsRNA sensors of the innate immune system, RIG-I and melanoma differentiation-associated gene 5 (MDA5). Upon binding to their dsRNA ligand, RIG-I/MDA5 are activated and interact with their adaptor protein mitochondrial antiviral signaling (MAVS) to induce the RLR signaling pathway and subsequent type I IFN production [14,15]. Although both KSHV and EBV are DNA viruses, infection with these viruses has been reported to induce RLR signaling pathways because their infection leads to the production of virus- and host-derived RNAs with double-stranded structures, such as miRNAs, circular RNAs, and long noncoding RNAs. In addition to RLRs, KSHV and EBV infection can also be regulated by other RNA binding proteins involved in innate immunity, such as protein kinase R (PKR) and TLRs 3, 7 and 8 [12].

In this review, we summarize the RNA sensors regulating gammaherpesviruses and their signaling pathways involved in the innate immune system. During KSHV and EBV replication, the viral- and host-derived immunostimulatory RNAs are abundant and some of them are able to bind and activate cellular RNA sensors, leading to the induction of the RNA-activated innate immune response to control viral replication and infection. While the host cell uses these RNA sensors to limit virus infection, KSHV and EBV utilize various viral and/or host proteins or noncoding RNAs to evade innate immune responses and facilitate viral replication. 

## 2. RIG-I-Like Receptors 

### 2.1. Gammaherpesviruses Activate the RLR Signaling Pathway

**Figure 1:**
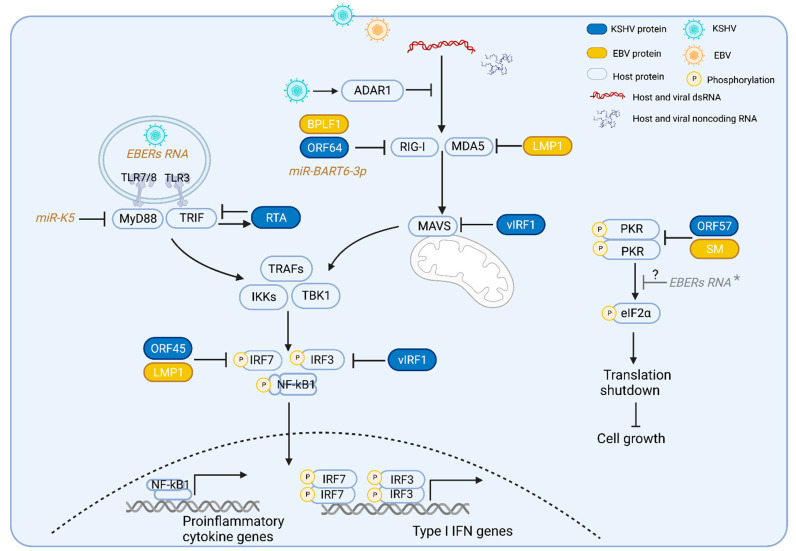
Gammaherpesviruses activate and evade the RNA-induced innate immune pathways. TLRs 3, 7, and 8 detect RNA in the endosome and RLRs (RIG-I and MDA5) detect dsRNA in the cytoplasm. During KSHV and EBV infection, TLRs 3, 7, and 8, and RLRs are activated by host or viral RNAs; activated TLR3 and TLR7/8 recruit and induce their downstream adaptor proteins TRIF and MyD88, respectively. Activated RLR induces the adaptor protein MAVS. These adaptor proteins subsequently recruit and induce common downstream proteins TNF receptor-associated factors (TRAFs) and TANK-binding kinase 1 (TBK1), leading to the phosphorylation and activation of the transcription factors interferon-regulatory factor 3 (IRF3), IRF7 and NF-κB that results in the production of type I interferons and other proinflammatory cytokines. Activation of TLR3 signaling induces KSHV RTA expression, which in turn promotes TRIF degradation. KSHV miRNA K5 blocks MyD88. KSHV ORF64, EBV BPLF1, and EBV miBART-3p inhibit RIG-I activation. EBV LMP1 reduces both RIG-I and MDA5 expression. KSHV vIRF1 inhibits the activation of RLRs’ adaptor protein, MAVS, and TLR3-mediated activation of IRF3. Upon binding to dsRNA, PKR undergoes autophosphorylation and becomes an activated kinase to phosphorylate a key translation initiation factor (eIF2α), inducing the shutdown of global protein synthesis and inhibiting cell growth. KSHV ORF57, EBV SM or EBV noncoding RNA EBERs interact with PKR and inhibit PKR activation. * EBER: the role of EBERs in PKR phosphorylation is unclear due to conflicting reports.

RLRs are RNA sensors localized in the cytosol which include RIG-I, MDA5 and the laboratory of genetics and physiology (LGP2). All three RLRs share similar RNA binding domains, including the conserved DExD/H helicase domain and C-terminal domain (CTD) [16]. The N-termini of RIG-I and MDA5 have two additional tandemly linked caspase activation and recruitment domains (CARDs) that mediate the activation of adaptor protein MAVS [14,17,18]. Upon activation, MAVS recruits and activates downstream proteins, TNF receptor-associated factors (TRAFs), IκB kinase (IKK) and TANK-binding kinase 1 (TBK1). These subsequently activate transcription factors, nuclear factor-κB (NF-κB), interferon regulatory factor 3 (IRF3) and IRF7, which induce the expression of Type I IFN genes and proinflammatory cytokine genes (Figure 1) [14]. As LGP2 lacks the CARD domain, it does not directly activate MAVS and is considered a regulator of RIG-I and MDA5 [19]. Although RIG-I and MDA5 induce the same downstream signaling pathways upon RNA binding, they have distinct preferences for the RNA duplex structure they bind to. RIG-I recognizes dsRNA with a triphosphate group at its 5′ end (5′ppp) and RIG-I can also bind to long noncoding RNAs without these ends [20,21], while MDA5 senses long RNA duplexes (>4 kb) independent of 5′ppp [22].

Both EBV and KSHV infections trigger the RLR signaling pathway through viral/ host RNA binding to the RNA sensors, RIG-I and MDA5. West et al. first reported that dsRNA is induced during KSHV lytic replication by demonstrating its accumulation in reactivating cells using a dsRNA-specific antibody J2 [23]. Further studies revealed the binding of multiple RNA transcript fragments induced by KSHV lytic reactivation to RIG-I, such as ORF2_543561–43650_, ORF8_10420–10496_ and the repeat region LIR1_119059–119204_, through high-throughput sequencing of RNA isolated by the immunoprecipitation approach [24]. In addition to the recognition of KSHV viral dsRNA by RIG-I, many host RNAs have been identified to be bound to RIG-I and MDA5 during KSHV lytic reactivation [25]. Host vault RNAs (vtRNAs) are the most highly enriched RIG-I bound RNAs in KSHV-infected cells [25]. vtRNAs are expressed in unstressed cells in a non-immunostimulatory state as their 5′ppp ends are removed by the cellular dual specificity phosphatase 11 (DUSP11) [26,27]. However, KSHV lytic reactivation reduced the expression of DUSP11 and increased the RIG-I activating 5’ppp-vtRNAs [25]. Furthermore, depletion of RIG-I, MDA5 or their adapter MAVS individually enhances KSHV replication during viral lytic reactivation and primary infection [23,25], suggesting that the KSHV-induced RIG-I/MDA5-mediated RLR signaling pathway restricts viral infection.

EBV-encoded RNA1 (EBER1) and EBER2 are small noncoding RNAs that are transcribed by host RNA polymerase III (Pol III) and are abundantly expressed in latent EBV-infected cells. In spite of EBER1 and EBER2 being short RNAs of 167 and 172 nucleotides, respectively, both have secondary structures consisting of intermolecular base-pairing and several stem-loops that can trigger RLR sensing [28,29]. EBERs are primarily found in the nucleus, although there is some evidence of their presence in the cytoplasm and their interaction with cytosolic proteins [30]. Inhibition of polymerase III activity suppresses the expression of EBERs and decreased EBER-induced type I interferon production [31]. EBERs are able to interact with RIG-I and activate RIG-I-mediated type I IFNs and ISGs production, and either EBER could trigger type I IFN responses independently [31,32]. EBERs also induce IL-10 production which is dependent on the activation of RIG-I where depletion of RIG-I or expression of RIG-I lacking in the CARD domain blocks EBER-induced IL-10 expression [33]. This indicates that EBERs are recognized by RIG-I and activate downstream signaling to induce type I IFN and other cytokines in EBV-infected cells, and this activity involves the CARD domain of RIG-I. Thus, EBV infection stimulates expression of immunostimulatory RNA substrates for dsRNA sensors that trigger type I IFN and cytokine production.

### 2.2. Gammaherpesvirues Evade the RLR Signaling Pathway by Utilizing Both Viral and Host Proteins

KSHV and EBV deploy multiple viral proteins to disrupt RLR activation during de novo infection and lytic reactivation in order to efficiently evade the antiviral response and establish their life cycle. The protein homologs BPLF1 and ORF64 are viral deubiquitinating enzymes (DUBs) of EBV and KSHV, respectively, that target the RLR sensor RIG-I [34,35,36]. RIG-I is subject to K63-polyubiquitination by ubiquitin ligases, including tripartite motif protein 25 (TRIM25), Riplet, Mex-RNA binding family member C (MEX3C), TRIM4 and TRIM21 [37,38]. This K63-polyubiquitination of the RIG-I CARD domain is essential for activating adaptor protein MAVS and recruiting downstream signaling molecules [37,39]. Both ORF64 and BPLF1 have been shown to decrease RIG-I ubiquitination, leading to reduced RIG-I activation and suppression of downstream innate immune responses [40,41]. EBV BPLF1 promotes the dimerization and autoubiquitination of TRIM25, which leads to impaired RIG-I ubiquitination [40]. KSHV also utilizes the viral interferon regulatory factor 1 (vIRF1) to target MAVS and block RLR signaling. vIRF1 is recruited to the mitochondria and inhibits MAVS aggregation during virus replication that in turn negatively regulates the MAVS-mediated antiviral responses and promotes KSHV replication [42]. Additionally EBV-encoded latent membrane protein 1 (LMP1) degrades RIG-I and MDA5 by recruiting E3 ubiquitin ligases to induce the proteasomal degradation of RIG-I and MDA5 [43]. Furthermore, EBV-encoded microRNA miBART6-3p targets the 3′ untranslated region (UTR) of the RIG-I mRNA, resulting in the decreased expression of RIG-I-induced interferon and interferon-stimulated genes (ISGs) [44]. In addition, since Pol-III-transcribed EBERs are able to activate the RIG-I sensing pathway as described above, the EBV lytic protein replication and transcription activator (Rta) interacts with Pol III to suppress the expression of EBERs and other immunogenic small RNAs [45]. Thus, gammaherpesviruses not only directly inhibit the activation of proteins involved in the RLR signaling pathway but also decrease the availability of RLR ligands induced during KSHV and EBV reactivation and replication.

In addition to utilizing virus-derived proteins and noncoding RNAs, EBV and KSHV also hijack host proteins to evade the RLR signaling pathway. ADARs are RNA-editing enzymes that bind to dsRNA and convert adenosine to inosine in dsRNA. There are three members of the human ADAR family, designated ADAR1 (ADAR), ADAR2 (ADARB1), and ADAR3 (ADARB2), where ADAR1 is responsible for the majority of the A-to-I editing activity in mammalian cells [46]. Widespread A-to-I editing of both the host and viral transcripts has been observed in KSHV-infected cells, and the A-to-I editomes are further expanded during KSHV lytic reactivation [47]. The A-to-I editing of the induced dsRNAs by KSHV infection prevents them from being recognized and detected by RLRs within the cell. Thus, in the absence of ADAR1, these KSHV-induced dsRNAs lacking A-to-I editing are exposed to and recognized by MDA5/RIG-I to stimulate the RLR antiviral signaling pathway, leading to the increased induction of IFNs, and resulting in the inhibition of KSHV lytic replication [48]. A-to-I editing also affects latent EBV viral infection. EBV pri-miR-BART6, targeted by the Dicer enzyme of the mammalian RNA-induced silencing complex (mRISC), modulates the EBV latency state through the control of viral gene expression. A-to-I editing of pri-mi-BART6 suppresses its targeting by Dicer which leads to viral lifecycle transitions to either type III latency or lytic reactivation [49]. In addition to pri-mi-BART6, A-to-I editing has also been found in EBV pri-miR-BART3, pri-miR-BART8 and pri-miR-BART11, as well as the KSHV K12/Kaposin transcript [50,51]. Furthermore, EBV A-to-I hyperedited *OriP* transcripts can bind to ADAR1 and promote EBV viral lytic replication [52]. Hence, KSHV and EBV not only utilize their own proteins or RNAs, but also usurp cellular proteins to escape the innate immune response. 

## 3. Toll-Like Receptors

TLR3 (Toll-like receptor 3) is a dsRNA sensor that localizes on the endosomal membrane and recognizes dsRNA structures in the lumen of the endosome. Upon binding to dsRNA, TLR3 recruits and activates its adaptor protein TIR-domain-containing adapter inducing IFN-β (TRIF), leading to the induction of its downstream signaling pathway to promote the production of inflammatory cytokines and type I IFN [53]. Primary infection of monocytes with KSHV upregulates and activates the TLR3 pathway, which induces the expression of downstream cytokines IFN-β and CXCL10 [54]. However, KSHV-encoded vIRF1 blocks TLR3-mediated IFN-β production through decreased phosphorylation and the nuclear translocation of IRF3 [55]. vIRF1 can also block TLR3-induced innate immunity via the ubiquitin-like protein interferon-stimulated gene 15 (ISG15), which is important for the stabilization of IRF3 in the innate antiviral response. vIRF1 can bind to ISG15 E3 ligase to decrease the TLR3-induced ISG15 conjugation of proteins and reduce the protein level of cellular IRF3 to blunt the type I IFN response [56,57]. Another KSHV lytic protein replication and transcription activator (RTA) can also interfere with the TLR3 sensing pathway. It has been shown that RTA is induced upon activation of the TLR3–TRIF signaling pathway and the induced RTA in turn degrades the TLR3 adaptor, TRIF, by shortening the half-life of the TRIF protein to block the innate immune response [58,59]. KSHV RTA also acts as a ubiquitin E3 ligase to promote the polyubiquitination and degradation of IRF7, which decreases IRF7-mediated IFN production [60]. Similarly, KSHV ORF45 blocks IRF7 phosphorylation and inhibits IFN induction [61,62]. For EBV, TLR3 was detected to be highly expressed in EBV-associated nasopharyngeal carcinomas (NPC) cell lines and clinical specimens [63]. Importantly, EBV noncoding RNA EBERs induce an inflammatory response via TLR3 in NPC cells, which promotes tumor growth (Figure 1) [64]. Additionally, EBV-infected cells can release EBERs complexed with a cellular EBER-binding protein, La, which can be recruited into exosomes via endocytosis and subsequently activate TLR3 and the downstream signaling pathways through NF-κB and IRF3 transcription [65,66]. Moreover, the EBV protein, LMP1, can cooperate with TLR3 to activate NF-κB-mediated downstream pro-inflammatory signaling and rescue TLR3 activation-induced apoptosis in several NPC cell lines [67]. LMP1 can also facilitate the phosphorylation and nuclear translocation of IRF7 [68]. Finally, EBERs also play a pivotal role in enhancing the LMP1-induced NF-κB-mediated inflammatory response [64]. As is the case with all pathogens, innate immune defenses to EBV and KSHV are triggered during infection but proteins encoded by these viruses are subsequently able to dampen these innate immune responses. In the case of TLR3 activation by EBV and KSHV infection, viral proteins and noncoding RNAs counteract the innate immune response.

TLRs 7 and 8 are located on the endosomal membrane and recognize single-stranded RNA (ssRNA) with guanosine and uridine-rich sequences [69]. Upon binding to RNA molecules, TLRs 7 and 8 recruit and activate the adaptor protein MyD88 (myeloid differentiation primary response 88) to induce the downstream signaling pathway, leading to inflammatory cytokine and type I IFN production [70,71]. EBV infection induces the expression of TLR7 and TLR8, as well as downstream mediators in the TLR7 signaling pathway [72,73]. In addition, EBV infection also enhances B cell proliferation in a TLR7-dependent manner [72,73,74,75,76]. These reports indicate that EBV infection is able to activate TLR7/8 and induce the downstream signaling pathway. However, another report shows that TLR7/8 activation concurrent with EBV infection can also inhibit B cell proliferation [77]. Interestingly, TLR7 signaling can also induce the expression of LMP1, a key latent transforming protein of EBV, in type III latency EBV-infected cell lines [78]. Furthermore, EBV EBERs induce TLR7-driven IFNα production [79]. For KSHV, TLR7/8 stimulation by its agonists or TLR7/8 stimulation by vesicular stomatitis virus infection promotes lytic reactivation from latency in PEL cell lines [80]. Downstream of TLR 7/8 are the signaling cascade mediators, Interleukin-1 receptor (IL-1R)-associated kinase 1 (IRAK1) and MyD88, that are targeted by KSHV. KSHV miRNAs miR-K9 and miR-K5 reduce the TLR7/8 agonist-induced NF-κB activation and IFNα expression by targeting IRAK1 and MyD88 [81].

## 4. Protein Kinase R

Protein kinase R (PKR) is a dsRNA-dependent protein kinase that is induced by interferons. dsRNA binding induces PKR dimerization and autophosphorylation, leading to its activation as a kinase. Activated PKR phosphorylates the α subunit of eukaryotic translation initiation factor 2 (elF2α), causing global translation shutdown and cell growth inhibition. In addition, PKR activation also elicits the formation of stress granules (SGs), which are able to trigger the host cell antiviral response and inhibit virus production [82,83]. To counteract this response, KSHV and EBV utilize viral proteins to disrupt the PKR signaling pathway and allow for productive viral infection. KSHV RNA binding protein, ORF57, interacts with two dsRBD (double-stranded RNA binding domains) motifs of PKR to block its dimerization and kinase activation, thus preventing eIF2α phosphorylation and SG formation, and allowing KSHV gene expression and viral production [84]. The Epstein–Barr virus (EBV) SM protein is a post-transcriptional regulator of viral gene expression that exports unspliced viral mRNA from the nucleus to the cytoplasm for mRNA translation [85]. Along with aiding viral mRNA export, the SM protein binds to dsRNA and associates physically with PKR, which prevents PKR activation [85]. EBV noncoding RNA, EBER1, has also been implicated in blocking PKR activation by binding to the dsRBD of PKR through its stem loop structure, inhibiting its phosphorylation and preventing interferon-induced apoptosis of Burkitt lymphoma cells [86,87]. However, another report showed that EBER1 blocked interferon-induced apoptosis independent of PKR phosphorylation (Figure 1) [88]. This suggests that other mechanisms might be at play downstream of PKR activation to block interferon-induced apoptosis in EBER-expressing cells [88]. Additionally, PKR also plays an important role in regulating LMP1-induced NF-κB nuclear translocation and cytokine production such as that of interleukin-6 (IL-6) and IL-10 [89]. Thus, PKR is targeted by both EBV and KSHV for modulating the innate immune response.

## 5. RNA Modifications Impact the Immune Response to KSHV and EBV

Within a cell, the addition of chemical moieties to RNA, i.e., RNA modifications, can affect its stability, secondary structure, and protein binding activity, which impact the recognition of RNA molecules by innate immune sensors, including RLRs, TLRs, and PKRs in the immune signaling pathway [16]. More than one hundred different types of RNA modifications have been identified in RNA molecules with A-to-I editing, *N*^6^-methyladenosine (m^6^A), 5-methylcytosine (m^5^C), and pseudouridylation comprising some of the most well-studied RNA modifications [90]. 

In addition to the increase in A-to-I editing of viral and host RNAs during KSHV and EBV infection, as described above, infections with these two viruses also lead to enhanced N6 methyladenosine (m^6^A) modifications in both viral and cellular RNA molecules [91,92]. m^6^A modification is carried out by the m^6^A methyltransferases (METTL3/14/16), also called writers of the m^6^A [90]. The modified m^6^A RNA is recognized by m^6^A-binding proteins, also known as readers in the RNA epigenetic pathway, such as YTHDF1/2/3, YTHDC1/2, and HNRNPA2B1, and can be removed by demethylases called erasers such as FTO and ALKBH5 [90]. Various m^6^A readers, writers and erasers regulate the lytic reactivation of KSHV [91,92,93,94]. The KSHV-encoded protein, SOX, affects overall mRNA stability by inducing RNA decay, while the cytokine interleukin 6 (IL-6) is able to escape the degradation by SOX [95]. The m^6^A modification of IL-6 mRNA and the recruitment of reader YTHDC2 to the mRNA is required for protecting IL-6 from degradation induced by the SOX protein [96]. Additionally, the m^6^A modification of cellular mRNA, *GPRC5A*, enhances its RNA stability causing an increase in GPRC5A protein which subsequently inhibits NF-κB signaling pathway and promotes KSHV lytic reactivation [97]. 

Similar to KSHV, many m^6^A editing proteins influence EBV latency and lytic replication [98,99]. Importantly, EBV lytic reactivation enhances m^6^A modification of the cellular transcripts DTX4 and TYK2 mRNA by decreasing the expression of demethylase ALKBH5 that results in the attenuation of innate immunity and facilitates EBV lytic reactivation [100]. 

Recently, another abundant RNA modification, pseudouridylation, has been identified in EBER2 where pseudouridylation of EBER2 is important for the viral lytic replication for EBV [101]. Pseudouridine is also found in KSHV RNAs, and pseudouridylation synthases (PUS) are enzymes that catalyze the conversion of uridine to pseudouridine in RNA molecules, and exhibit a proviral role during KSHV lytic reactivation [102]. Thus, pseudouridylation is important for viral lytic replication for both EBV and KSHV. How this modification controls the innate immune response to these viruses remains yet to be determined.

## 6. Conclusions and Perspective

We have discussed how KSHV and EBV infection activates several cellular RNA sensors, including RIG-I, MDA5, PKR, TLR3, TLR7 and TLR8 to induce the downstream signal pathways and innate immune responses (Figure 1 and Table 1). In addition to deciphering how each RNA sensor may influence gammaherpesviral infections, it is also important to study the differences between the mechanisms of these RNA sensors and whether they have overlapping roles in activating the innate immune response. Another important consideration is the interplay amongst the various RNA sensing pathways during viral infection. While activated PKR phosphorylates eIF2α to shutdown global protein translation, it can also trigger the RLR signaling pathway to induce the production of type I interferons (IFNs) and other pro-inflammatory cytokines. In addition, activation of RIG-I and MDA5 converges on the utilization of the adaptor protein MAVS to activate the RLR signaling pathway, while a certain MAVS isoform negatively regulates TLR3 signaling [103]. These findings demonstrate how various RNA sensing pathways may be interdependent in driving innate immune responses. Moreover, the cGAS-STING DNA sensing pathway can also be activated by the RLR pathway and promotes crosstalk between the DNA and RNA sensing pathways [104,105]. Thus, understanding the complex interplay among the various nucleic acid sensing pathways in the context of DNA viral infections is important to fully understand the mechanisms of how gammaherpesvirus infections occur and persist in host.

Although various RNA sensing pathways are induced during KSHV and EBV infection, only a small number of RNA ligands recognized by these RNA sensors have been identified and further studied. One of the most well-known transcripts are EBV-noncoding EBERs, which can activate several RNA sensors including TLR3, TLR7 and RIG-I. Thus, EBERs can be utilized as an RNA ligand to learn about the crosstalk among the RNA signaling pathways. This could also contribute to clarifying the question of whether the inhibition of IFN-induced apoptosis by the EBERs is dependent on the activation of PKR. EBERs are expressed in the latent state and act as a ligand for RNA sensing. Therefore, EBV must employ various strategies to avoid the EBER activation of RNA sensing pathways to prevent apoptosis and maintain viral latent infection within host cells. In addition to LMP1 and miBART6 promoting the degradation of RLRs to decrease IFN induction, EBERs themselves play a role in the inhibition of IFN-induced apoptosis, but the mechanism is still unclear. Hence, studying the structure, binding affinity and functions of the RNA ligands that are induced during KSHV and EBV infection will advance our understanding of virus–host interactions during KSHV/EBV infection.

Recently, it has been shown that many KSHV and EBV RNAs undergo RNA modifications including methylation, A-to-I editing and pseudouridylation, which affect RNA stability, secondary structure, and protein association as well as the recognition of RNAs by RNA sensors [90,92,98,99,100,101,102]. For example, A-to-I editing of viral and host RNA can help KSHV evade the RLR sensing pathway and facilitate viral infection [47,48]. While m^6^A modifications of viral and host RNAs influence KSHV and EBV infections, future work should be geared towards determining how the various m^6^A readers, writers and erasers mechanistically alter various m^6^A sites in both viral and cellular mRNAs to modulate the innate immune response and whether they exhibit functional redundancy. Overall, elucidating how KSHV and EBV activate and escape the RNA-mediated innate immune response may contribute to a better understanding of the virus-associated pathologies and provide new insights into developing effective strategies against viral infection.

## Figures and Tables

**Table 1:** RNA sensors modulate the innate immune response to gammaherpesvirus infection.

RNA Sensors	Nucleic Acid Binding Domain	Known Functions	Interaction with Viral Proteins/ncRNAs	Impact on EBV/KSHV
RIG-1	Helicase domain, C-terminal domains	Antiviral innate immune activation	KSHV ORF64, EBV BPLF1, EBV LMP1	Inhibits viral replication
MDA5	Helicase domain, C-terminal domains	Antiviral innate immune activation	EBV LMP1	Inhibits viral replication
TLR3	N-terminal Ectodomain (ECD)	Antiviral innate immune activation	EBV EBERs	Induces KSHV RTA expression
TLR7/8	leucine-rich repeat (LRR) domain	Antiviral response, Tumor immunity Regulation	KSHV miR-K9 and miR-K5	Enhances B-cell proliferation, Induces KSHV lytic reactivation
PKR	dsRBDs	Protein translation shutdown	KSHV ORF57, EBV SM	Inhibits viral gene expression
ADAR1	dsRBD, DNA binding domain	dsRNA editing	KSHV K12 transcript, EBV pri-miBARTs	Facilitates KSHV lytic reactivation. Influences the function of EBV pr-miBART6

## Data Availability

Not applicable.
